# Expression of Molecular Markers of Resilience against *Varroa destructor* and Bee Viruses in Ethiopian Honey Bees (*Apis mellifera simensis*) Focussing on Olfactory Sensing and the RNA Interference Machinery

**DOI:** 10.3390/insects14050436

**Published:** 2023-05-03

**Authors:** Haftom Gebremedhn, David Claeys Bouuaert, Michel Asperges, Bezabeh Amssalu, Lina De Smet, Dirk C. de Graaf

**Affiliations:** 1 Laboratory of Molecular Entomology and Bee Pathology, Ghent University, 9000 Ghent, Belgium; haftomgebremedhn.ngussie@ugent.be (H.G.); david.claeysbouuaert@ugent.be (D.C.B.); dirk.degraaf@ugent.be (D.C.d.G.); 2 Tigray Agricultural Research Institute, Mekelle P.O. Box 492, Ethiopia; 3 Centrum Voor Milieukunde, University of Hasselt, 3590 Diepenbeek, Belgium; 4 Holeta Bee Research Center, Holeta P.O. Box 22, Ethiopia

**Keywords:** honey bee, resilience, molecular markers, olfactory sensing, RNA interference, viruses, deformed wing virus, *Varroa destructor*

## Abstract

**Simple Summary:**

Globally, honey bees are exposed to many challenges, such as the *Varroa destructor* mite and various viruses, which lead to massive losses. It is generally believed that African honey bees are more resilient and better able to cope with these stressors. This study examined some molecular markers that may be associated with this resilience. Higher resistance to the varroa mite could be related to better olfactory sensing. Higher gene expression levels of the odorant binding protein OBP14 in the antennae of Ethiopian honey bees suggest that reproducing mites might be better detected and cleaned. Resistance or tolerance to viruses could be attributed to a better functioning antiviral RNAi system. Several genes involved in this pathway are upregulated and are positively correlated with the viral load in honey bees. Both mechanisms may contribute to the resilience of African bees to varroa infestation and viral infection.

**Abstract:**

*Varroa destructor* mites and the viruses it vectors are two major factors leading to high losses of honey bees (*Apis mellifera*) colonies worldwide. However, honey bees in some African countries show resilience to varroa infestation and/or virus infections, although little is known about the mechanisms underlying this resilience. In this study, we investigated the expression profiles of some key molecular markers involved in olfactory sensing and RNA interference, as these processes may contribute to the bees’ resilience to varroa infestation and virus infection, respectively. We found significantly higher gene expression of the odorant binding protein, OBP14, in the antennae of Ethiopian bees compared to Belgian bees. This result suggests the potential of OBP14 as a molecular marker of resilience to mite infestation. Scanning electron microscopy showed no significant differences in the antennal sensilla occurrence and distribution, suggesting that resilience arises from molecular processes rather than morphological adaptations. In addition, seven RNAi genes were upregulated in the Ethiopian honey bees and three of them—*Dicer-Drosha*, *Argonaute 2*, and *TRBP2*—were positively correlated with the viral load. We can conclude that the antiviral immune response was triggered when bees were experiencing severe viral infection and that this might contribute to the bees’ resilience to viruses.

## 1. Introduction

Globally, high losses of managed honey bee colonies have been reported, especially in the Northern Hemisphere, resulting in severe economic losses [1,2,3,4]. These colony losses have been attributed to both biotic and abiotic stressors. Among the biotic factors, the *Varroa destructor* mite and infection by the RNA viruses are the most important [1,2,5]. Both the varroa-mite and RNA viruses like deformed wing virus (DWV), Lake Sinai virus, acute bee paralysis virus, chronic bee paralysis virus, sacbrood virus, and black queen cell virus have been reported in different parts of Africa [6,7,8,9,10]. Unlike the Western bees, honey bees in some African countries such as South Africa [11], Ethiopia [12,13], Kenya [14], and Uganda [15] suffer less from mite infestations. Moreover, severe colony losses due to virus infections have not been reported in Africa [16]. The resilience to bee-related diseases of African honey bees is worth studying, as it can provide insights into how honey bees defend themselves against their main disease-causing agents.

Honey bees’ resilience to varroa infestations has been studied for over 35 years. Indeed, in 1984, Ruttner reported that honey bees in Uruguay, denied any treatment, developed strategies to control the varroa-mite population [17]. Later on, the mechanisms that honey bees rely on to fight the mite became evident and include grooming behavior [18,19], hygienic behavior [18,19,20,21], and suppressed mite reproduction [22,23]. Grooming is the detection, aggressive removal, and destruction of adult mites from the bodies of adult bees [24], whereas hygienic behavior (now termed ‘Varroa Sensitive Hygiene’ (VSH)) is a three-step process consisting of detection, opening, and removal of infested pupae by worker bees [25]. Suppressed mite reproduction represents the inability of the mites to reproduce in the brood cell. There was some confusion about this term as it was initially given to the social hygienic behavior of removing the reproducing mites [26,27], but now it is narrowed to the physiological inability of mites to produce viable offspring in the brood cells [28,29]. In the past decades, honey bee populations that have developed some kind of resilience against varroa-mites have been found on a global scale as result of selective breeding [30,31,32] and natural selection [33,34,35,36,37,38,39].

There is overwhelming evidence that the antennae of worker bees hold the key to VSH [40]. First, evidence was obtained by electroantennography [41,42] and the proboscis-extension reflex tests [42], where hygienic bees responded better to the odors associated with infested broods. This was later reinforced by the finding of differentially expressed genes related to olfaction in transcriptome [40,43] and proteome studies [44,45] of mite-resistant stocks. Moreover, the search for genetic markers for selection based on (genome-wide) linkage mapping pointed to a crucial role of olfactory sensing [46,47,48,49]. Recently, a whole exome sequencing approach identified the cytoskeletal motor protein dynein beta chain (only found in mechano- and chemosensory neurons) as one out of eight variants associated with the SMR trait [28], demonstrating that the key role of the antennae in varroa-resistance goes beyond VSH.

A protein family that often recurs in the aforementioned studies is the odorant binding proteins (OBPs). These proteins deliver hydrophobic airborne molecules to olfactory receptors but most probably also function as general carriers in other developmental and physiological processes [50]. Honey bees have a relatively small set of only 21 OBPs [50]. Six have been associated with varroa-resistance: OBP1 [48,51], OBP2 [52], OBP14 [40,52], OBP16 [45], OBP17 [52], OBP18 [45,52]. In Canada, two of these, OBP16 and OBP18, were included in a marker-assisted selection approach for disease resistance traits of honey bees [53]. Recently it was shown that DWV-A altered the olfactory sensitivity and the expression of odorant binding proteins in the antennae of honey bees, which may adversely affect the aroma perception affecting their performances [54]. In line with these findings, the present gene expression study of targeted genes involved in olfactory sensing and/or associated with varroa-resistance in the antennae of worker bees aimed to find molecular markers associated with the low mite infestation levels of Ethiopian bees [13]. In addition, as a pilot experiment, scanning electron microscopy (SEM) was used to identify morphological differences between the antennae of varroa-resistant Ethiopian bees (*A. m. simensis*) and those of their Belgian varroa-sensitive counterparts (*A. m. carnica*).

The resilience to virus infections in honey bees is much less investigated. Only recently, the ‘suppressed in ovo virus infection’ trait was found to be a good indicator of the colonies’ overall resistance to virus infections [55]. Here, the term ‘resistance’ should be interpreted as the ability to limit parasite burden, whereas ‘tolerance’ means the ability to limit the harm caused by a given burden [56]. Tolerance to virus infections has been reported in honey bee populations in the Old World that were subjected to natural selection, both in Europe [57,58] and Africa [16,59], including Ethiopia [59]. Although the underlying mechanism is still unclear, there are indications that viral diversity and virulence play an important role. The entrance of the varroa-mite has influenced the viral diversity and dynamics. Indeed, the era prior to the introduction of the varroa-mite was characterized by low prevalence, low viral load, and high strain diversity of DWV, which was completely turned upside down once *V. destructor* was established [60]. The mite acts as a vector for bee viruses and narrows the viral landscape [61]. Whether the dominance of a certain genotype leads to the health or death of the colony is much debated and appears to be context dependent [60,62,63,64]. In the studied region, the dominance of the B-type of DWV (DWV-B) was found to coincide with the lack of clinical symptoms and thus could at least partially explain the observed virus tolerance [59]. It has been suggested that after the initial period of a selective sweep, varroa-adapted DWV strain diversification can be re-established, which matches well with the relatively large diversity of DWV-B found in Ethiopian bees. This process of diversification is possibly driven by the selection of rare lineages that can evade the genotype-specific antiviral defenses based on RNA interference (RNAi) [65]. RNAi antiviral immunity in insects is a post-transcriptional gene-silencing mechanism directed by small interfering RNAs (siRNAs). The pathway is activated when double-stranded RNA (dsRNA), produced as an intermediate of viral replication, is recognized and diced into siRNAs (18–24 bp) by the RNase type III enzyme Dicer2 (Dcr2). These siRNAs are then incorporated into an RNA-Induced Silencing Complex (RISC) and will serve to target viral RNA genomes for degradation [66,67]. We have previously shown that key components of the RNAi machinery in virus-sensitive Belgian honey bees are suppressed when they experience high virus infection levels [68]. As bees in Ethiopia express some sort of virus tolerance, it seemed most unlikely that the same relationship between virus load and antiviral immunity would exist. To demonstrate this, we extended our focus to a whole-body gene expression study targeting selected genes of the RNAi machinery, comparing Ethiopian with virus-susceptible Belgian honey bees.

## 2. Experimental Section

### 2.1. Study Area and Honey Bee Samples

The Ethiopian honey bees examined in this study were collected from the Tigray National Regional State as part of a previous metagenomic study on *A. m. simensis*. Detailed information on sample collection, transport, and storage can be found in Gebremehdn et al. (2020) [59]. Samples were collected (with the owners’ permission) from 20 bee colonies spread over 10 apiaries between August and October 2017 (i.e., in the active season). Guard bees were collected late at night at the hive entrance using a torch light [59]. Samples were stored at −24 °C at the Laboratory of Biotechnology, Mekelle Agricultural Research Center, before shipping to Ghent University while keeping them frozen (on dry ice), where they were stored at −80 °C until further testing. In Mekelle, the bees could not be stored at −80 °C due to the lack of a freezer. In [59], we could show that the isolated RNA from these samples was of high quality, and this is thus usable for expression studies. Belgian honey bees, *A. m. carnica*, were taken from the apiary of the University of Ghent and served as controls. The samples of honey bees were age and season-matched. The Belgian control bees, 44 in total, were sampled between June and July 2019 from 8 different colonies. We do realize that the environment is completely different, but in Ethiopia, we were not able to sample varroa-sensitive honey bees as we believe that all African bees are more resilient.

The gene expression study of the antennae was performed on samples from the districts Degua Temben, Hawzen, and Mekelle (i.e., one colony/district) and on three Belgian control colonies. From each colony, the antennae from four worker bees were pooled. 

The gene expression study of the RNAi machinery was performed on whole body samples from seven colonies originating from the Ganta Afeshum, Kilte Awlaelo (2 colonies), Gulo Mkada, Degua Temben, Mekelle and Atsby Wonberta districts (3 worker bees/colony). These districts belong to similar agroecological zones (high to mid-highlands only). Colonies were selected based on DWV titers (low and high) as determined by RT-qPCR and NGS (i.e., number of reads) from our previous metagenomic study [59]. The groups (i.e., bees with high and low viral load) were determined based on K-Means clustering analysis (for details, see Statistical analysis).

### 2.2. Homogenization and RNA Extraction

The antennae of worker bees were removed using fine scissors, pooled (4 pairs of antennae/colony in each vial), and homogenized in the presence of 500 μL Qiazol (Qiagen, Hilden, Germany), 0.25 mL zirconia beads and 3 metal beads using the TissueLyser machine at 3500 rpm for 1 minute. The homogenate was frozen at −80 °C for 1 h to enhance cell lysis, and after thawing, 100 μL chloroform was added and vortexed for 15 s followed by incubation at room temperature (RT) for three minutes. After centrifuging the samples at 12,000× *g* for 15 min, the upper phase was carefully removed. Total RNA was extracted using the RNeasy lipid tissue mini kit (Qiagen), according to the manufacturer’s specifications. The RNA was eluted in 30 μL of RNase-free water. The extracted RNA template was immediately stored at −80 °C until further use.

Whole-body RNA extracts were made from individual adult bees. Each bee was homogenized in 1 mL Qiazol (Qiagen), 0.25 mL zirconia beads, and 3 metal beads using a TissueLyser machine at 3500 rpm for 1 minute. After 5 min incubation at RT, 200 μL chloroform was added, and the samples were mixed by vortexing for 15 s and incubated at RT for an additional 2–3 min. After 15 min centrifugation at 12,000× *g*, the upper phase was carefully transferred to a new tube, and the total RNA was extracted using the RNeasy lipid tissue mini kit (Qiagen), including on-column DNase digestion, according to the manufacturer’s guidelines. The RNA was eluted in 50 μL RNase-free water. 

### 2.3. cDNA Synthesis

cDNA was synthesized using the Thermo Scientific RevertAid First Strand cDNA Synthesis Kit (Thermo Scientific, Waltham, MA, USA) following the manufacturer’s protocol. cDNA from the antennae was synthesized using 1 µL of random hexamer primers (50 ng/µL), 10 µL RNA template, and 1 µL of nuclease-free water, whereas cDNA from whole body extracts was synthesized from 5 µL RNA template. After incubating at 65 °C for 5 min, 4 µL of 5× reaction buffer, 1 µL of Ribolock RNase inhibitor, 2 µL of 10 mM dNTP mix, and 1 µL of Revert aid H Minus M-MuLV Reverse transcriptase was added to each sample and incubated at 25 °C for 5 min, 42 °C for 60 min and 70 °C for 5 min. The resulting cDNA was stored immediately at −20 °C.

### 2.4. Viral Load Quantification

The DWV loads were determined in samples from antennae and individual bees (whole-body) by RT-qPCR using the SYBR^®^ Green fluorescence. For the antennae, the RT-qPCR reaction was performed in a total volume of 15 µL, consisting of 7.5 µL platinum SYBR Green qPCR SuperMix-UDG (Thermo Scientific), 0.03 µL forward primer (100 µM), 0.03 µL reverse primer (100 µM), 6.44 µL of DEPC-treated water and 1 µL cDNA template. For the individual bees, the RT-qPCR reaction was performed in a total volume of 25 µL. All reactions were performed in triplicate with the following PCR conditions: 50 °C for 2 min; 95 °C for 2 min, followed by 40 cycles at 95 °C for 15 s, 58 °C for 20 s, and 72 °C for 30 s. At the end of the program, a melt curve analysis to verify the presence of the desired amplicon (temperature increase of 0.5 °C for 5 s over a range from 65 °C to 95 °C) was performed. A virus-free (negative control) sample was included in each assay.

DWV loads in each sample were quantified using absolute quantification methods based on standard curves obtained through serial fivefold dilutions of known amounts of the amplicon. Viral load was expressed as viral copy number per bee. This was calculated by multiplying the number of copies obtained in RT-qPCR by the different dilution factors. The viral copy number/bee for the antennae and individual bees was obtained by multiplying the reported qPCR copy number values by the dilution factor of 60 and 200, respectively.

### 2.5. Gene Expression Analysis

The primers used for RT-qPCR in the gene expression study of the antennae and the individual bees are given in Tables S1 and S2, respectively. The primers related to olfactory sensing or antennae functioning were designed in the present study using the Primer3 software (https://bioinfo.ut.ee/primer3/ accessed on 4 November 2019). Primer specificity was determined by a melt curve analysis, confirming the amplification of a single product [69]. The amplification efficiencies of all the primers were validated based on a standard curve that was constructed using serial five-fold dilutions of pooled cDNA.

The expression of selected genes in antennae and individual bees (whole-body) was determined by RT-qPCR as described for viral load quantification (see previous paragraph), except that in the whole-body reactions, the cDNA samples were diluted 5 times. The thermocycler program for the antennae samples was a two-step amplification protocol: 50 °C for 2 min, 95 °C for 2 min, and 95 °C for 30 s, 60 °C for 30 s for 40 cycles. For the individual bee samples, this was: 50 °C for 2 min, 95 °C for 2 min and 95 °C for 20 s, and 60 °C for 40 s for 40 cycles. The RT-qPCR assays of the (pooled) antennae samples were performed in triplicate, whereas those of the (individual) whole-body samples were performed in duplicate. Negative controls were included in each assay. Each target gene was assayed for all the samples on a single plate. 

In order to normalize the real-time PCR data, we included reference genes in our assays [70]. For the antennae samples, we used β-actin as the reference gene in accordance with [71,72]. For the individual bee samples, we re-examined a set of six known reference genes (eIF3-S8, RPS5, RPL8, Enolase, MGST, and GADPH) in order to determine the most stable ones [68]. Therefore, we used the geNormPLUS algorithm within the qBasePLUS environment (Biogazelle NV, Zwijnaarde, Belgium) with default settings [68]. These analyses revealed that the optimal number of reference genes was two, with *eIF3-S8* and *GADPH* as the most stable ones (Figure S1).

### 2.6. Scanning Electron Microscopy

Antennae were carefully excised from the antennal sockets with fine forceps under a stereo microscope. Fixation occurred in 2% glutaraldehyde in washing buffer (0.05 M sodium cacodylate buffer pH 7.3 with 0.15 M saccharose) for 24 hours. Samples were washed twice for 10 min and dehydrated by an ethanol serial solution (30%, 50%, 70%, 95% to 100%) with a 30 min interval between solutions. Then they were critically dried in Polaron and sputter coated with gold/palladium. Finally, they were mounted on the frontal or caudal side on sticky tape and examined by a Hitachi S-570 SEM set at 20 kV. In total, 12 antennae were examined, equally distributed over antennae coming from Ethiopian and Belgian worker bees (both left and right antennae). Micrographs of the antenna as a whole, the antennomeres, flagellomeres, and sensillae were taken. The classification of the sensillae was in accordance with previously described morphological criteria [73,74], and the subdivision into olfactory and non-olfactory sensillae was as described in Jung et al. [72,75].

### 2.7. Statistical Analysis

A *t*-test was used to compare the expression of olfactory genes in the antennae of Ethiopian and Belgian honey bees. Each group contained a pool of 8 antennae from 3 different colonies. The difference between the two groups was analyzed after the Bonferroni correction for multiple comparisons in qbase software. 

For the correlation study between DWV load and expression levels from the RNAi- related genes, the load of DWV were log-transformed before the statistical analysis to conform with the assumptions to the normality of the data. A *p*-value less than 0.05 was considered significant. The association between the normalized expression of the RNAi-related genes and the load of DWV was analyzed using the Pearson correlation in the case of Ethiopian bees. While in the case of Belgian bees, the association between the RNAi-related genes expression and the load of DWV was analyzed using the Spearman correlation since the normality assumptions were not fulfilled. In this analysis, all tested samples were included. 

In addition to these analyses in which DWV load was treated as a continuous variable, the samples in the case of Ethiopian bees were clustered into two categories: bees with high (4.11 × 10^12^ virus copy number per bee, *n* = 9) and low (7.69 × 10^7^ virus copy number per bee, *n* = 12) viral load using the K-means clustering of the Elbow method (Figure S4). The expression of each target gene in bees with high and low viral loads was compared using the independent *t*-test. 

The expression of RNAi-related genes between Ethiopian and Belgian bees was also compared using the independent *t*-test. 

The Pearson correlation, Spearman correlation, K-means clustering using the Elbow method, and independent *t*-test were analyzed using the SPSS (version 27), IBM Corp., Armonk, NY, USA. Graphs related to trend analysis, box plots, and stability analysis of the reference genes were developed using Microsoft Excel and R (version 3.6.1) using the packages ggplot2 (https://www.R-project.org (v4.1.2; R Core Team 2021, accessed on 4 November 2019)) and qbase software, respectively.

## 3. Results

### 3.1. Gene Expression and DWV Loads in the Antennae

We found significantly higher gene expression of *OBP14* (*p* = 0.0198) in the antennae of Ethiopian bees when compared to Belgian bees (Table 1). The gene expression of *vitellogenin* (*Vg*) was also substantially higher, although it was not significant. These differences were considerable, with fold changes (f.c.) close to 20 (*Vg*: f.c. = 20,384; *OBP14*: f.c. = 16,144). The expression of *Emp24* was considerably lower but not significant in Ethiopian bees when compared to the Belgian (f.c. = 0.096).

Further, no statistical differences were found in the DWV loads of the antennae between the Ethiopian and the Belgian bees. Bees were screened for the presence of DWV complex and not for the presence of a specific genotype. The measured DWV titers were high, with an average DWV load/bee of 3.0 × 10^9^ and 9 × 10^9^ for Ethiopian and Belgian bees, respectively.

### 3.2. RNAi Activation and DWV Loads in Whole Bees

**Figure 1:**
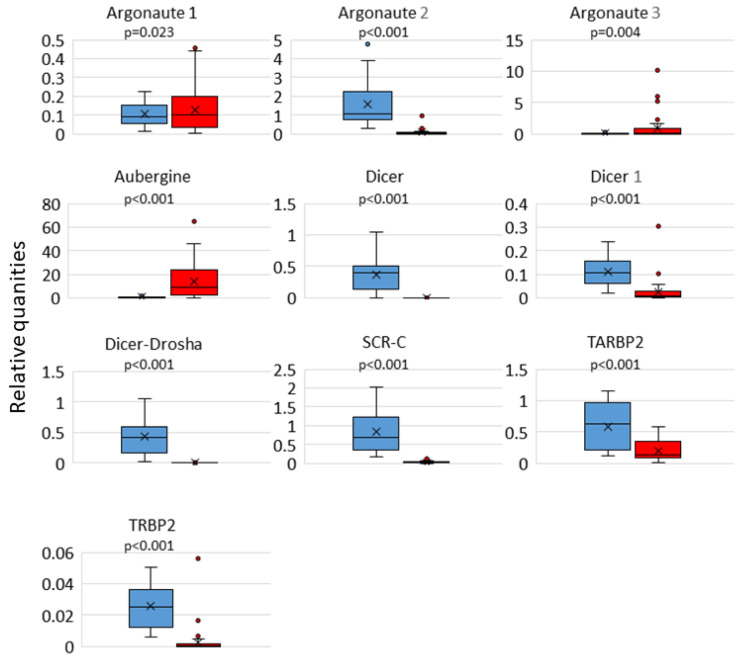
Box plot of normalized RNAi-related gene expression in bees. The expression of each target gene was normalized using two reference genes (*eIF3-S8* and *GADPH*). Normalized gene expression (ΔΔCq) is the relative quantity of the target gene normalized to the quantities of the reference genes. In blue: Ethiopian honey bees (21 colonies); in red: Belgian honey bees (44 colonies). The means are marked by a cross (×) on the box plots.

The gene expression levels of 7 out of 10 key genes of the RNAi machinery (*Argonaute 2*, *Dicer*, *Dicer 1*, *Dicer-Drosha*, *SCR-C*, *TARBP2*, and *TRBP2*) were higher in the Ethiopian bees when compared with Belgian bees. Only *Argonaute 1*, *Argonaute 3*, and *Aubergine* showed lower expression levels in Ethiopian bees (Figure 1).

**Figure 2:**
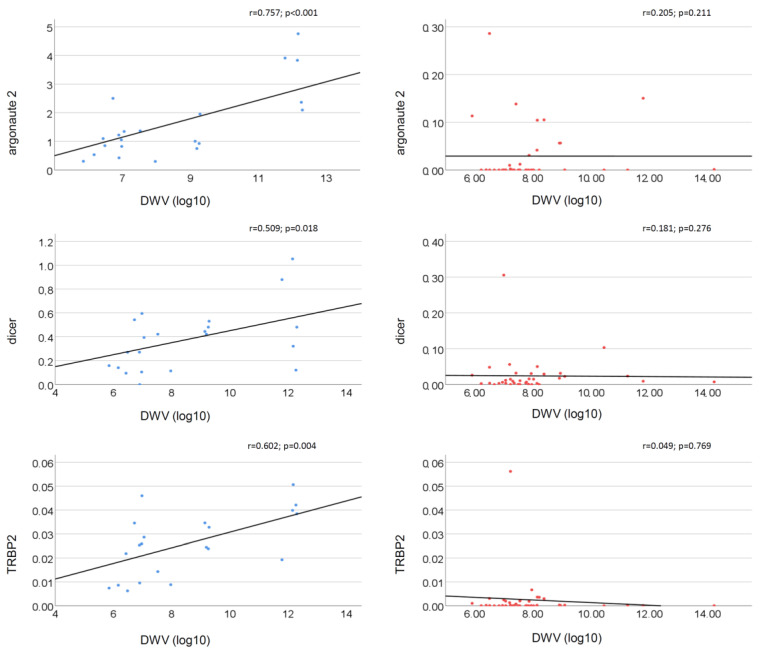
Correlation between the DWV load (log) and the normalized expression levels of RNAi-related genes in bees. In blue: Ethiopian honey bees (21 colonies); in red: Belgian honey bees (44 colonies).

The relationship between the DWV load and gene expression levels also shows remarkable differences between the Ethiopian and Belgian bees. When considering the viral load as a continuous variable, the DWV load was positively correlated with the expression of *Argonaute 2* (r = 0.757; *p* < 0.001), *Dicer* (r = 0.509; *p* = 0.018) and *TRBP2* (r = 0.602; *p* = 0.004) for the Ethiopian honey bees (Figure 2). However, this correlation was missing in the Belgian bees (Figure 2). None of the other genes showed a significant correlation with the DWV loads (Figure S2).

Using the K-means clustering, the Ethiopian bees were clustered in two groups (Figure S4) with low (average 7.69 · 10^7^ virus copy number/bee, N = 11) and high DWV loads (average 4.11 · 10^12^ virus copy number/bee, N = 9). When considering the viral load as a categorical variable DWV loads, the target genes *Dicer-Drosha*, *TRBP2,* and *Argonaute 2* were significantly (*p* < 0.05) upregulated with fold changes of 3.101, 1.973 and 2.348 respectively in the highly infected Ethiopian bees (Table 2). Dicer did not show a significantly different gene expression, albeit showing the highest fold change (f.c. = 3.857).

The association level of expression among the components of the RNAi machinery was studied by means of correlation analysis (Table 3 and Table 4). In the case of Ethiopian bees, we observed similarities in the expression patterns between the target genes (i.e., positive correlations) (Table 3), while in the Belgian bees, some negative correlations were observed (Table 4).

### 3.3. External Morphology of the Antennae

**Figure 3:**
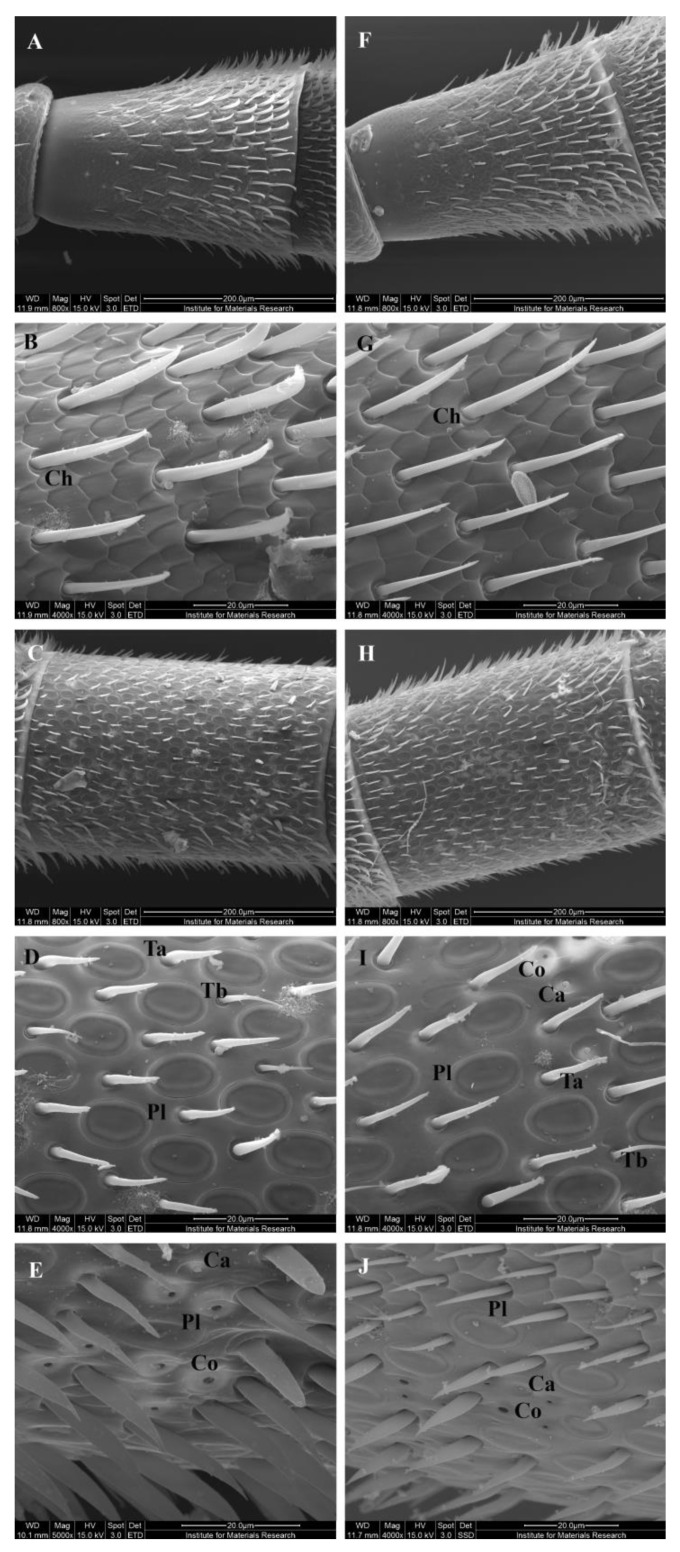
Scanning electron micrographs of the sensillae on the antennae. (**A**,**F**) Flagellomere 1; (**B**,**G**) Details of flagellomere 1; (**C**,**H**) Flagellomere 3; (**D**,**I**) Detail flagellomere 3 anterior side; (**E**,**J**) Detail flagellomere 3 posterior side. Ta, sensillae trichodea type a; Tb, sensillae trichodea type b; Pl, sensillae placodea; Co, sensillae coelonica; Ca, sensillae campaniformia. On the left: Ethiopian honey bees; on the right: Belgian honey bees.

The antenna of the honey bee and most insects is morphologically differentiated into three parts: scape (proximal antennomere), pedicel (second antennomere), and flagellum, the latter consisting of 10 flagellomeres (Figure S3). Flagellomeres 1 and 2 have no olfactory sensillae and are mainly covered with sensilla chaetica. From flagellomeres 3 onwards, multiple olfactory sensillae were found: sensillae trichodea type a and b, sensillae placodea, and sensillae basiconica thick. Sensillae placodea were the most abundant on the anterior side, whereas sensillae coelonica and sensillae campaniformia (both non-olfactory sensillae) were rather found on the posterior side. The overall occurrence and distribution of sensory organs were similar in Ethiopian and Belgian honey bees (Figure 3).

## 4. Discussion

In this study, we were seeking molecular markers that could explain the promising resilience of Ethiopian honey bees against varroa mites and deformed wing viruses reported in our previous studies [13,59]. The present study focused on target genes that emerged from previous studies. The 10 genes related to olfactory functioning have been identified in different genome/exome-wide association studies (*OBP1* [48] and *dynein* beta chain [28]), genome-wide transcriptome studies (*OBP3*, *OBP14* [40,52], *OBP16* [52], *GB43812*, and *vitellogenin* [40]), targeted gene expression studies (*OBP3*, *OBP16*, and *OBP18* [76]) and proteome studies (OBP16 [45], OBP18 [45,77], Emp24, and Cop-gamma [77]), all in the context of defensive mechanisms against the varroa-mite The current expression study was performed on guard bees which implies that we may have missed the expression changes caused by direct interaction of the varroa mites with his host as Varroa mites prioritize to parasitize nurse bees in their phoretic stage [78]. However, the samples guard bees were exposed to mites in their earlier live stage, which implies that we are still able to detect markers that are responsible for the defense mechanisms against the varroa mites. The 10 genes related to the RNAi machinery came from our previous work [68], which demonstrated that the key components of RNAi were down-regulated in Belgian honey bees with high viral load. In fact, in this study we wanted to extend this study by measuring the gene expression level over a wider gradient of DWV loads and compare it to populations less affected by viral infections, i.e., the Ethiopian *simensis* honey bee. The same comparison was made between Belgian and Ethiopian bees regarding the expression of olfactory genes. Preferably we should have made the comparison with varroa-sensitive bees from Ethiopia, but these were not available in the studied population. This can be seen as a weakness in our experimental design, given the fact that climate, vegetation and beekeeping practices in Africa and Belgian are almost opposite to each other. However, since our study was focused on genes for which increased expression has been repeatedly shown to be associated with defensive traits against the varroa-mite, we thought making the comparison with the Belgian varroa-sensitive population is reasonable. 

Our study revealed significantly higher expression levels of *OBP14* in the antennae of Ethiopian guard bees. *Vitellogenin* was also higher but not significantly different expressed in the Ethiopian bees. *OBP14* is one of the odorant-binding proteins already discussed in the introduction. Vitellogenin is a 180 kDa glycolipoprotein synthesized in the fat body and released to the hemolymph [79]. It is best known as a yolk protein produced by the queens, but in addition to that, it plays a role in worker bees’ behavioral traits such as nursing, foraging onset, and foraging bias, and in survival traits such as oxidative stress resilience, cell-based immunity and longevity [80]. *OBP14* and *vitellogenin* were also found to be upregulated in RNA-seq analyses of the antennae from VSH bees of New Zealand [40]. These two genes even belonged to the top 10% most upregulated genes of the total of 258 differentially expressed genes in VSH when compared to non-VSH bees. They were among the 57 genes that were differentially expressed between foragers and nurse bees: *vitellogenin* was over-expressed in nurse bees, whereas *OBP14* was over-expressed in foragers [40]. *OBP14* was also upregulated in varroa-tolerant compared to varroa-susceptible colonies that emerged from the Saskatraz project in Canada [52]. However, in these populations, higher expression was found in pupae when infested with mites, and this upregulation was the highest in pupae of varroa-tolerant colonies [52]. The high expression level of *OBP14* is in line with earlier observations that the VSH trait—which is strongly expressed in Ethiopian bees [13]—is an odor-guided behavior. The fact that its upregulation was found independently in three studies, each in a different continent (the present study and [40,52]), underlines the potential of *OBP14* as a molecular marker of resilience to mite infestation. The high expression levels of *vitellogenin* call for a more critical assessment because of the multitude of physiological processes in where vitellogenin is involved in. Honey bee workers’ behavioral switch from nurse bees to foragers is accompanied by an increase in the juvenile hormone titer and a decrease in the vitellogenin protein level [81]. On top of that vitellogenin expression is nutritionally-regulated [82] and was found to go down in collapsing colonies [83] and to be high in winter survivors [84]. 

Antennae of worker bees are endowed with many sensillae [75,85,86], which play a critical role in the initiation of varroa-sensitive hygienic behavior [40]. Here, we compared the occurrence and distribution of antennal sensilla in Ethiopian and Belgian bees but found no noteworthy differences. Gramacho et al. [87] reported that the number of antennal plate organs is not greater in hygienic Africanized bees when compared to non-hygienic Africanized bees, which is in line with our findings. It seems that hygienic behavior and, thus, antennal function is rather determined at the molecular level than at the morphological level.

The present study found a strong antiviral immune response in Ethiopian honey bees and positive correlations between the components that contribute to this. Moreover, several RNAi-related genes are also positively correlated with the DWV load. *Dicer* and *Argonaute 2* were significantly upregulated in Ethiopian bees with high DWV loads, which corresponds with the findings of Galbraith et al. [88], who studied the global gene expression (and DNA methylation) associated with acute Israeli acute paralysis virus (IAPV) infection. They found that several transcriptionally regulated genes were associated with viral response pathways in insects, including the RNAi pathway, with *Dicer* and *Argonaute 2* (*Ago2*) as the most pronounced upregulated genes. Therefore, we can further validate them as molecular markers in breeding programs toward viral-resistant honey bee strains. All genes involved in the siRNA pathway showed higher expression levels in the Ethiopian bees. The expression levels of Aubergine and Argonaute 3 were lower, but both are Piwi proteins and function in the piRNA pathway [89]. It was previously shown that the piRNA pathway does not contribute to antiviral defense in honeybees [90], which is in line with our findings. That all involved genes from the siRNA pathway are upregulated strongly suggests that this pathway is part of the antiviral defense mechanism of honey bees. The rationale behind this could be that higher exposure to viruses results in a triggered antiviral immune response which may contribute to the bees’ resilience against pathogens. However, the comparison with the Belgian bees is remarkable and gives the impression that their anti-viral immunity is disturbed. Overall the expression level of key genes of the RNAi machinery is low, some components are negatively correlated, and a correlation with the DWV load is lacking. It is completely in line with our earlier observation, that was then hypothetically explained by the occurrence of viral suppressors of RNAi [68]. The observation in Belgian bees is comparable with the study of Ryabov et al. [91], in which no upregulation from *Argonaute* and *Dicer* in DWV-infected pupae could be detected. 

Mondet et al. [40] found lower DWV loads in the antennae of hygienic bees compared to those of non-hygienic bees [40] and hypothesized that DWV infections could affect the antennal function. Higher loads of DWV were also reported in the antennae of DWV-symptomatic bees compared to the antennae of bees from the same colony emerging without symptoms of DWV [92]. In the present study, the presence of DWV was demonstrated in the antennae of both the Ethiopian and Belgian worker bees, though the virus loads were not significantly different. Thus, our observations are more in line with [93], who revealed similar DWV loads in the antennae of hygienic and non-hygienic bees, suggesting that the difference in hygienic behavior (i.e., whether hygienic or non-hygienic) may not be related to the level of viral load in the antennae of honey bees. However, it has recently been reported that DWV-A infections in honey bees are able to down-regulate the expression of *OBP2*, *OBP5*, *OBP11*, and *OBP12* leading to the disturbance of their aroma perception, and affecting their performance in tasks carried out in and outside of the colony [54]. The expression profile of *OBP14*, however, was not examined in that study.

## 5. Conclusions

Expression profiling of some key molecular markers involved in antiviral responses and olfactory sensing identified *Dicer*, *Argonaute 2*, and *OBP14* as promising markers for resilience in honey bees and suggested that both mechanisms may contribute to the resilience of Ethiopian honey bees against Varroa and DWV.

## Figures and Tables

**Table 1:** The relative gene expression of selected genes in the antennae of Ethiopian bees compared to Belgian bees. Antennae from 4 worker bees were pooled for three Ethiopian and three Belgian colonies. The statistical analysis was performed using a *t*-test.

Target	*p*-Value	f.c.
*OBP14*	0.0198	16.144
*Vg*	0.201	20.384
*GB43812*	0.201	2.436
*OBP16*	0.214	0.3
*Dynein*	0.214	7.61
*OBP18*	0.250	2.11
*OBP3*	0.260	5.521
*Emp24*	0.563	0.096
*Cop-gamma*	0.563	0.826
*OBP1*	0.563	1.274

Target genes in alphabetic order: *Cop-gamma*, coatomer subunit gamma isoform; *Dynein*, dynein beta chain, ciliary; *Emp24*, transmembrane emp24 domain-containing protein; *OBP1*, odorant binding protein 1; *OBP3*, odorant binding protein 3; *OBP14*, odorant binding protein 14; *OBP16*, odorant binding protein 16; *OBP18*, odorant binding protein 18; *Vg*, vitellogenin; *p*-value is multiple testing corrected; statistically significantly different when *p* < 0.05; f.c. is a fold change which measures the expression of a target gene in nominator relative to denominator subgroup.

**Table 2:** The relative expression of RNAi genes in bees with high and low viral load (DWV). The relative gene expression of each target gene was normalized with two reference genes (*eIF-S8* and *GAPDH*). Mean is the expression of each target gene relative to the reference genes; ratio is a fold change that measures the expression of a target gene in bees with high viral load relative to the expression of the target gene in bees with low viral load; high and low indicates honey bee groups with high and low viral load, respectively.

Target	DWV Load	Mean	N	Ratio (High/Low)	95% Value ci Low	95% Value ci High	*p*-Value
*Dicer-Drosha*	High	1.863	9	3.101	1.408	6.827	0.034
	Low	0.601	11				
*TRBP2*	High	1.453	9	1.973	1.212	3.212	0.034
	Low	0.737	11				
*Argonaute 2*	High	1.599	9	2.348	1.258	4.381	0.034
	Low	0.681	11				
*SRC-C*	High	1.406	9	1.858	1.046	3.299	0.074
	Low	0.757	11				
*Argonaute 3*	High	1.479	9	2.038	1.033	4.021	0.074
	Low	0.726	11				
*Aubergine*	High	1.465	9	2.002	1.1020	3.929	0.074
	Low	0.732	11				
*Dicer 1*	High	1.349	9	1.725	0.951	3.129	0.093
	Low	0.783	11				
*Dicer*	High	2.101	9	3.857	0.857	17.355	0.093
	Low	0.545	11				
*Tarbp2*	High	1.304	9	1.62	0.846	3.105	0.144
	Low	0.805	11				
*Argonaute 1*	High	1.262	9	1.527	0.852	2.735	0.144
	Low	0.827	12				

**Table 3:** The Pearson correlation analysis among the expression level of the genes involved in the RNAi machinery in the case of Ethiopian honey bees. * and ** indicate correlation is significant at the 0.05 and 0.01 levels, respectively.

	*Argonaute1*	*Argonaute2*	*Argonaute3*	*Aubergine*	*Dicer*	*Dicer1*	*Dicer-Drosha*	*SRCC*	*Tarbp2*	*TRBP2*
*Argonaute1*	1									
*Argonaute2*	0.095	1								
*Argonaute3*	0.617 **	0.193	1							
*Aubergine*	0.6328 *	0.212	0.919 **	1						
*Dicer*	0.485 *	0.595 **	0.293	0.243	1					
*Dicer1*	0.728 **	0.103	0.657 **	0.678 **	0.422	1				
*Dicer-Drosha*	0.536 *	0.387	0.002	0.114	0.471 *	0.411	1			
*SRCC*	0.489 *	0.583 **	0.505 *	0.518 *	0.611 *	0.602 *	0.607 **	1		
*Tarbp2*	0.812 **	0.338	0.601 **	0.616 **	0.481 *	0.700 **	0.627 **	0.696 **	1	
*TRBP2*	0.345	0.614 **	0.106	0.193	0.431	0.160	0.352	0.229	0.300	1

**Table 4:** The Spearman correlation analysis among the expression level of the genes involved in the RNAi machinery in the case of Belgian honey bees. * and ** indicate correlation is significant at the 0.05 and 0.01 levels, respectively.

	*Argonaute1*	*Argonaute2*	*Argonaute3*	*Aubergine*	*Dicer*	*Dicer1*	*Dicer-Drosha*	*SRCC*	*Tarbp2*	*TRBP2*
*Argonaute1*	1.00									
*Argonaute2*	−0.588 **	1.00								
*Argonaute3*	−0.240	0.798 **	1.00							
*Aubergine*	0.573 **	−0.231	0.224	1.00						
*Dicer*	−0.449 **	0.602 **	0.568 **	0.119	1.00					
*Dicer1*	−0.499 **	0.195	0.026	−0.397 **	0.070	1.00				
*Dicer- Drosha*	−0.584 **	0.734 **	0.496 **	−0.307 *	0.656 **	0.249	1.00			
*SRCC*	0.132	0.447 *	0.743 **	0.511 **	0.372 *	−0.180	0.136	1.00		
*Tarbp2*	0.749 **	−0.506 **	−0.043	0.747 *	−0.007	−0.462 **	−0.437 **	0.259	1.00	
*TRBP2*	−0.543 **	0.614 **	0.187	−0.608 **	0.224	0.307 *	0.629 **	−0.213	−0.754 **	1.00

## Data Availability

The database used and/or analyzed during the current study is available from the corresponding author on request.

## Supplementary Materials

The following supporting information can be downloaded at: https://www.mdpi.com/article/10.3390/insects14050436/s1, Figure S1: Stability analysis of reference genes; Figure S2: Correlation between DWV load and gene expression level. Figure S3: Morphological organization of the honey bee antenna; Figure S4: The optimum number of clusters obtained by the k means cluster analysis using the elbow method. K, number of clusters. Table S1: Primers used in the gene expression study of the antennae; Table S2: Primers used in the gene expression study of the individual bees (whole body).
