# Supplementary material for: Expression of Molecular Markers of Resilience against Varroa destructor and Bee Viruses in Ethiopian Honey Bees (Apis mellifera simensis) Focussing on Olfactory Sensing and the RNA Interference Machinery
Source: Insects. 2023 May 3;14(5):436. doi: 10.3390/insects14050436 (PMC10231090; doi:10.3390/insects14050436)
Supplement: Supplementary file 1 [file insects-14-00436-s001.zip › insects-2320179-supplementary.pdf]

Supporting information

Figure S1. Stability analysis of reference genes. Stability analysis was performed on 6 reference genes using the geNormPLUS algorithm within the qBase PLUS environment.

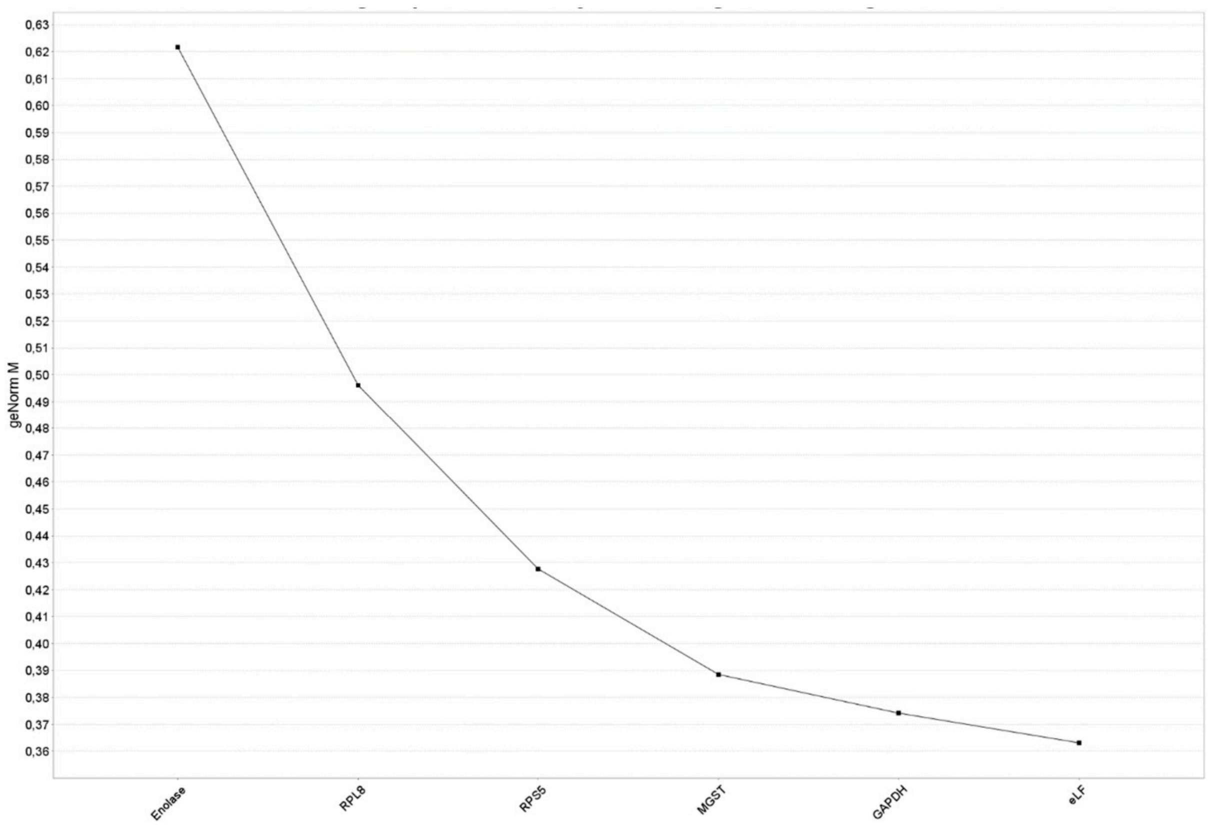

Figure S2. Correlation between DWV load and gene expression level. The expression of each target gene was normalized using two reference genes (eIF3-S8 and GADPH). Normalized gene expression (ΔΔCq) is the relative quantity of the target gene normalized to the quantities of the reference genes. In red: Ethiopian honey bees; in blue: Belgian honey bees.

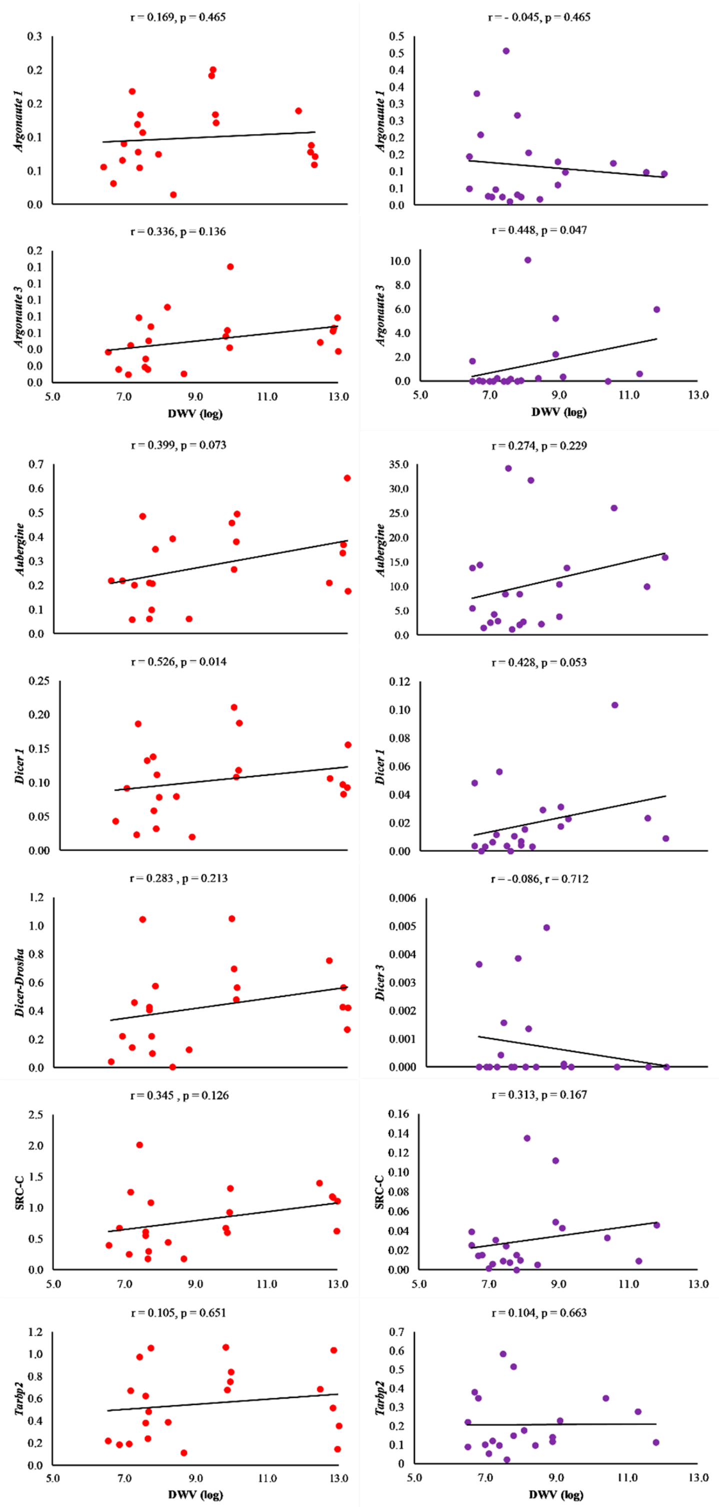

Table S1. Primers used in the gene expression study of the antennae. Abbreviations used: Vg, Vitellogenin; OBP1, odorant binding protein 1; OBP3, odorant binding protein 3; OBP14, odorant binding protein 14; OBP16, odorant binding protein 16; OBP18, odorant binding protein 18; Dynein, dynein beta chain, ciliary; Emp24, transmembrane emp24 domain-containing protein; Cop-gamma, coatomer subunit gamma isoform X1; Actin, Beta actin; F, Forward primer and R, Reverse primer.

Target genes	Primer	Primer sequence (5’- 3’)	Tm (°C)	Reference
**DWV**	DWV_F	GGT AAG CGA TGG TTT G	51.8	[85]
	DWV_R	CCG TGA ATA TAG GAG G	49.6	
**Vg**	Vit-F	ACG TAA TAA ATG CCG CCA AG	53.6	[68]
	Vit-R	TGC ATG TTG CTC TCC AAC TC	55.5	
**OBP1**	OBP1-F	AAA GGG CTC AGT CGG TCA TG	57.4	This study
	OBP1-R	CAG CCT GTT CTC GAT CCT CC	57.4	
**OBP3**	OBP3-F	ACG CCA ATG TGT CGA TAA TGC	56.1	This study
	OBP3-R	TGT CGC TGC TCT CTT CTT CG	57.1	
**OBP14**	OBP14-F	TGG TGC TCT GAC AAT CGA AG	54.9	This study
	OBP14-R	TCC ATG ACT GCT TTG ATT CCT	54.1	
**OBP16**	OBP16-F	GGC GAA ACT GGC ACT AGT CA	57.5	This study
	OBP16-R	TCG TCT AAA ACT GCT TGC ACA	54.8	
**OBP18**	OBP18-F	TAC GTG CTG TGG TGC CAA TT	57.5	This study
	OBP18-R	TTC CAC CAC CAT CGT CAT AGC CA	56.1	
**Dynein**	Dynein-F	CTC GGT GCT GAA CAT GGT CT	57.3	This study
	Dynein-R	GAG TCT GGC GTA ATC TCC CG	57.3	
**GB43812**	GB43812-F	ACC GAC TGC TTG GCT AAA GA	56.6	This study
	GB43812-R	GTC ACC GCA TCC ATC TTT CT	55.0	
**Emp24**	Emp24-F	GAA TGC ATA CGT GGC GCT G	57.2	This study
	Emp24-R	CCC ATT TTC ATG CCT GCT TCC	57.0	

**Cop-gamma**	Cop-gamma-F	TGT GAA GTC CCA TGT CCA CG	57.2	This study
	Cop-gamma-R	TCC TCG ACC TTG ATC AGC CA	56.0	
**Actin**	AmActine-F	TGC CAA CAC TGT CCT TTC TG		[84]
	AmActine-F	AGA ATT GAC CCA CCA ATC CA		

Figure S3. Morphological organisation of the honey bee antenna. Sc, scape; Ped, pedicel.

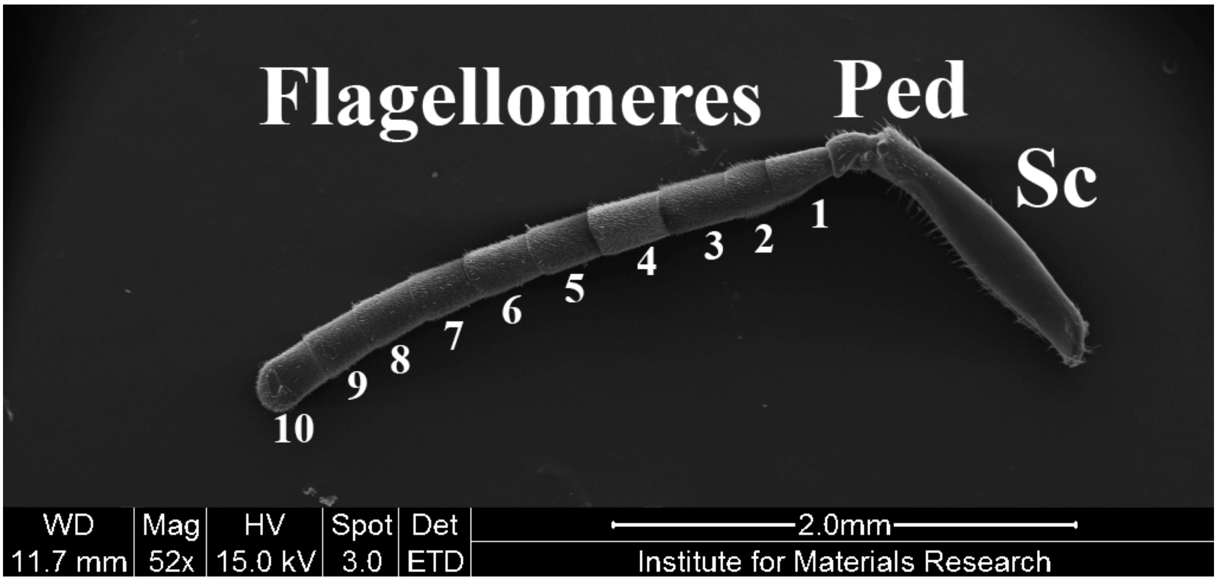

Figure S4. The optimum number of clusters obtained by the k means cluster analysis using the elbow method. K, number of clusters. The dashed straight line indicates the optimum number of clusters obtained, K = 2.

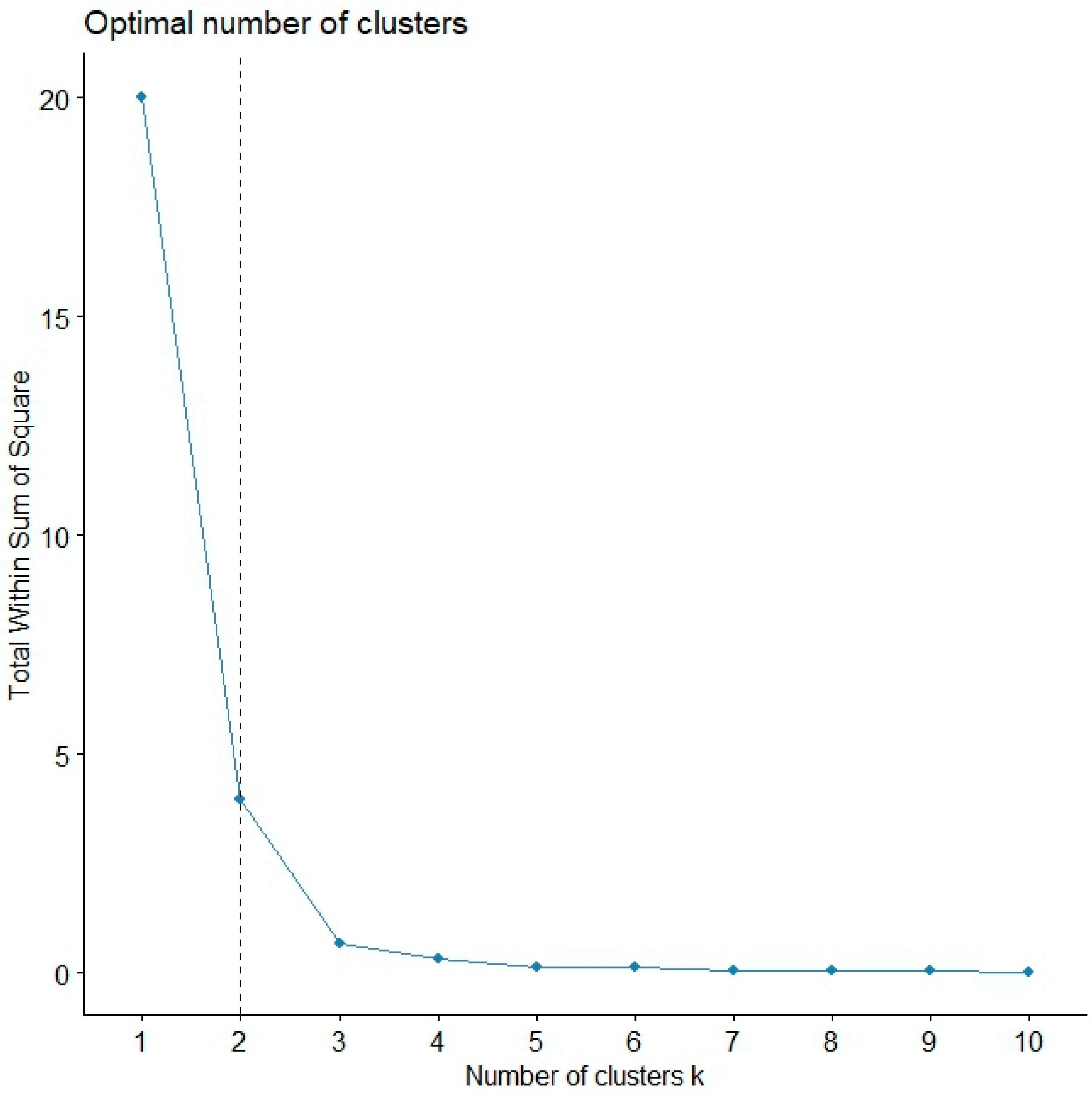

Table S2. Primers used in the gene expression study of the individual bees (whole body). Abbreviations used: DWV, Deformed wing virus; GAPDH, glyceraldehyde 3-phosphate dehydrogenase; MGST, microsomal glutathione S-transferase; elF, eukaryotic initiation factors; RPL8, ribosomal protein L8; RPS, ribosomal proteins of the small subunit; SRC-C, scavenger receptor class C; TARBP, TAR RNA-binding protein; TRBP, TAR RNA-binding protein; F, Forward primer; R, Reverse primer.

**Gene**	**Primer**	**Primer sequence (5'- 3')**	**Tm (°C)**	**Reference**
DWV	DWV_F	GGT AAG CGA TGG TTT G	51.8	[85]
	DWV_R	CCG TGA ATA TAG GAG G	49.6	
Argonaute 1	Amago1-F	AAC ATT TCG CGC TTC AGA TTA	53.1	[68]
	Amago1-R	TGC GCA AAA TAT TTG ATG TGA	52.5	
Argonaute 2	Amago1-F	AGC TAT TGC GCG CTT AGA	56.5	[68]
	Amago-R	GGT GCC CGC CTG TAC ATT	57.5	
Argonaute 3j	Amago1-F	GCA AGT TTG AGC GAC AGT ATT C	54.4	[86]
	Amago-R	ATA CAT CAC ATC GGG CAT TGG	55.3	
Aubergine	Amaub-F	TTA CCA ACG CCT CTC AAC CAAT G	57.6	[86]
	Amaub-R	AGA TAT ACC AAT TCG GCT TGA CCA G	56.6	
Dice	AmDicer2-F	AGC AGT AGC TGA TTG TGT	51.0	[68]
	AmDicer2-R	TCA GAA GCG CAA GGC ATG	56.1	
Dicer 1	AmDicer2-F	ACC AGA GGA TGC AGA AGT	57.4	[68]
	AmDicer2-R	TGT TAA CGC CTAC AGC TGT	56.4	
Dicer-Drosha	AmDicer2-F	TGC ACC TTC TTG GAT TCG	56.7	[68]
	AmDicer2-R	GCT GCA GTC CTA CGT AAT	55.7	
TRBP 2	AmPRM1-F	ACC AGC ATC ACC AGC TCA AT	56.9	[68]
	AmPRM1-R	TGT GGA GGT CCA ACA TCA CT	56.0	
SCR-C	AmSRCC-F	TAC GAG GCA GCA GCT ATG	57.0	[68]
	AmSRCC-R	TTG ATC ATC GTC GCT CTC	56.9	

TARBP2-like	AmTARBP2-F	TCA CCA GAT TCT CCA CTT CCA AA	56.1	[68]
	AmTARBP2-R	GGA GGC CAA TGA CGT GAC A	57.7	
eIF3-S8	AmlF3-F	TGA GTG TCT GCT ATG GAT TGC	54.9	[87]
	AmlF3-R	TCG CGG CTC GTG GTA	56.7	
RPS5	Am-RPS5-F	AAT TAT TTG GTC GCT GGA ATT	51.8	[88]
	Am-RPS5-R	TAA CGT CAA GCA GAA TGT GGTA	55.9	
RPL8	AmRPL8-F	TGG ATG TTC AAC AGG GTT CATA	54.0	[88]
	AmRPL8-R	CTG GTG GTG GAC GTA TTG ATAA	54.4	
Enolase	AmEnolase-R	GGT GAT GAA GGT GGT TTG C	54.6	[71]
	AmEnolase-F	GAT GCA GCA ACA TCC ATA CC	54.0	
MGST	AmMGST-F	TTG CTC TGT AAG GTT GTT TTGC	54.1	[89]
	AmMGST-F	TGT CTG GTT AAC TAC AAATCC TCC TG	54.8	
GADPH	AmGAPDH-F	GAT GCA CCC ATG TTT GTTG	52.8	[71]
	AmGAPDH-R	TTT GCA GAA GGT GCA TCA AC	54.0	
